# Laparoscopic lavage is superior to colon resection for perforated purulent diverticulitis—a meta-analysis

**DOI:** 10.1007/s00384-016-2636-0

**Published:** 2016-08-27

**Authors:** Eva Angenete, David Bock, Jacob Rosenberg, Eva Haglind

**Affiliations:** 1 Department of Surgery, Institute of Clinical Sciences, Sahlgrenska Academy at University of Gothenburg, SSORG - Scandinavian Surgical Outcomes Research Group, Sahlgrenska University Hospital/Östra, SE-416 85 Gothenburg, Sweden; 2 Department of Surgery, Herlev Hospital, University of Copenhagen, Herlev Ringvej 75, DK-2730 Herlev, Denmark

**Keywords:** Diverticulitis, Laparoscopy, Morbidity

## Abstract

**Purpose:**

Perforated diverticulitis often requires surgery with a colon resection such as Hartmann’s procedure, with inherent morbidity. Recent studies suggest that laparoscopic lavage may be an alternative surgical treatment. The aim of this study was to compare re-operations, morbidity, and mortality as well as health economic outcomes between laparoscopic lavage and colon resection for perforated purulent diverticulitis.

**Methods:**

PubMed, Cochrane, Centre for Reviews and Dissemination, and Embase were searched. Published randomized controlled trials and prospective and retrospective cohorts with laparoscopic lavage and colon resection as interventions were identified. Trial limitations were assessed using the Grading of Recommendations Assessment, Development and Evaluation (GRADE) methodology. Re-operations, complications at 90 days classified according to Clavien-Dindo and mortality were extracted.

**Results:**

Three randomized trials published between 2005 and 2015 were included in the analysis. The studies included a total of 358 patients with 185 patients undergoing laparoscopic lavage. At 12 months, the relative risk of having a re-operation was lower for laparoscopic lavage compared to colon resection in the two trials that had a 12 month follow-up. We found no significant differences in Clavien-Dindo complications classified more than level IIIB or mortality at 90 days.

**Conclusions:**

The risk for re-operations within the first 12 months after index surgery was lower for laparoscopic lavage compared to colon resection, with overall comparable morbidity and mortality. Furthermore, Hartmann’s resection was more costly than laparoscopic lavage. We therefore consider laparoscopic lavage a valid alternative to surgery with resection for perforated purulent diverticulitis.

## Introduction

In patients with diverticulosis, 4–7 % will develop the inflammatory condition diverticulitis [1, 2]. A serious complication to diverticulitis is a perforation of the colon followed by a septic condition which often requires emergency surgery [3]. The intra-operative findings in perforated diverticulitis can be classified according to Hinchey [4], where Hinchey grade III represents a perforation with purulent peritonitis and grade IV a perforation with fecal peritonitis. Traditionally, emergency surgery for perforated diverticulitis Hinchey grade III and IV has included resection of the diseased bowel segment with a colostomy (Hartmann’s procedure) or more recently sometimes with a primary anastomosis and temporary diverting loop ileostomy [5, 6]. The morbidity after emergency surgery for perforated diverticulitis is substantial with complication rates between 24 and 87 % [6–8], and complications are also common at the reversal of the colostomy after Hartmann’s procedure [9, 10]. In 2008, Myers et al. presented data from a cohort of patients where the surgical procedure consisted of a laparoscopic lavage in patients with perforated diverticulitis Hinchey grade III [11]. They reported a low morbidity of 4 %, indicating that this treatment could be superior to surgery with colon resection. However, Myers’ study was not a controlled trial, and the demography for patients not included in the study was not presented rendering it difficult to assess bias [11]. Five randomized trials have since started, and three have reported their primary endpoints, the DILALA trial [12, 13], the SCANDIV trial [14], and the LOLA trial [15]. The LAPLAND trial (NCT01019239) and the SIGMOIDITE (NCT01837342) have not yet reported any results. Furthermore, a recent health economic evaluation has looked at Hartmann’s resection versus laparoscopic lavage in the DILALA trial [16].

The aim of this systematic review and meta-analysis was to compare and to provide combined results on re-operations, morbidity, and mortality from studies comparing laparoscopic lavage with colon resection as the treatment for patients with perforated diverticulitis with purulent peritonitis.

## Materials and methods

A specified statistical analysis plan was developed before data extraction [17]. The study adhered to the Preferring Reporting Items for Systematic Reviews and Meta-Analyses (PRISMA) guidelines throughout the process of this systematic review and meta-analysis [18] and was registered at PROSPERO [19], CRD42016033126.

Published randomized controlled trials and prospective and retrospective cohorts comparing laparoscopic lavage with colon resection and a stoma (Hartmann’s procedure) or colon resection with primary anastomosis were identified. The outcomes were percentage of patients with one or more re-operations, with a complication of at least grade IIIB, classified according to Clavien-Dindo [20] and all-cause mortality. Studies were not included if they did not involve a comparison between the studied techniques, if they were a guideline or a systematic review.

A PICOS (participants, interventions, comparisons, outcomes, and study design) was constructed prior to database search. Patients: adult patients with acute diverticulitis requiring emergency surgery, Intervention: laparoscopic lavage, Comparison: colon resection including Hartmann’s procedure, Outcome: re-operations (percentage of patient with one or more and/or total number of) within 12 months; adverse events 30 and/or 90 days after index operation, Study design: cohort studies and randomized controlled studies. The following databases were then searched: PubMed, Cochrane, Centre for Reviews and Dissemination (CRD) and Embase using the time limit 10 years and language limit English, Norwegian, Danish and Swedish. The databases were accessed on the 29th of October and the 4th of November 2015. The following electronic search strategy was used in PubMED: (((((diverticulitis OR diverticular)) AND (lavage OR irrigation OR drainage)) AND (perforat* OR peritonitis OR acute OR complicated))) NOT (Editorial[publication type (ptyp)] OR Letter[ptyp] OR Comment[ptyp]) (((((diverticulitis OR diverticular)) AND (lavage OR irrigation OR drainage)) AND (perforat* OR peritonitis OR acute OR complicated))) NOT (Editorial[ptyp] OR Letter[ptyp] OR Comment[ptyp]) (((((diverticulitis OR diverticular)) AND (lavage OR irrigation OR drainage)) AND (perforat* OR peritonitis OR acute OR complicated))) NOT (Editorial[ptyp] OR Letter[ptyp] OR Comment[ptyp]) (Editorial[ptyp] OR Letter[ptyp] OR Comment[ptyp]) (((diverticulitis OR diverticular)) AND (lavage OR irrigation OR drainage)) AND (perforat* OR peritonitis OR acute OR complicated) (perforat* OR peritonitis OR acute OR complicated) (lavage OR irrigation OR drainage) (diverticulitis OR diverticular).

In Cochrane: (diverticulitis or diverticular: title (ti), abstract (ab), key word heading (kw) (Word variations have been searched)) (perforat* or peritonitis or acute or complicated: ti,ab,kw) (Word variations have been searched). A combination of the two above was also used.

In CRD: diverticulitis OR diverticular AND perforat* or peritonitis or acute or complicated.

In EMBASE: diverticulitis OR diverticulosis OR diverticulitis or diverticular. (title (ti), other term (ot), abstract (ab), keyword heading (kw) AND lavage or irrigation or drainage. ti,ot,ab,kw. OR peritoneum lavage/ OR colon lavage/ OR intestine lavage/ OR lavage/ OR stomach lavage/ AND perforat* or peritonitis or acute or complicated .ti,ot,ab,kw. OR peritonitis/. The searches included combinations of the above. Reference lists and bibliographical data of pertinent articles and systematic reviews were hand-searched for additional relevant articles.

Data on demographics, methods, results, and bias were collected by two independent reviewers. Any discrepancies were resolved by discussion among at least three authors. Trial limitations were assessed using the Grading of Recommendations Assessment, Development and Evaluation (GRADE) methodology [21]. Data were extracted from the selected articles using a pre-defined extraction form. For one study, information on follow-up time was missing [22]. The investigators were contacted by e-mail on two separate occasions to enable inclusion in the analysis but without response. The investigators for the included studies were not contacted for further data or confirmation.

Demography and extracted patient characteristics were number of patients, sex, age, body mass index (BMI), American Society of Anesthesiology (ASA) classification, previous diverticulitis, Hinchey grade, and previous abdominal surgery. Postoperative data extracted were re-operations, complications classified according to Clavien-Dindo [20], and all-cause mortality. Data were extracted by the author statistician (DB) and reviewed and quality-checked by three independent reviewers. No other studies were found in trial registries.

### Data analysis

Information on primary and secondary endpoints was presented for the individual studies with relative risk (laparoscopic lavage vs. colon resection (Hartmann’s procedure or resection with primary anastomosis)) with 95 % confidence interval in Forest plots. Where applicable, information from different studies was combined by a fixed effect model [23]. For sensitivity analysis, a random effect model was used [23]. Heterogeneity was assessed by I [2] and Cochran’s Q [24]. Statistical analyses were performed using the R package metafor [25].

## Results

**Fig. 1:**
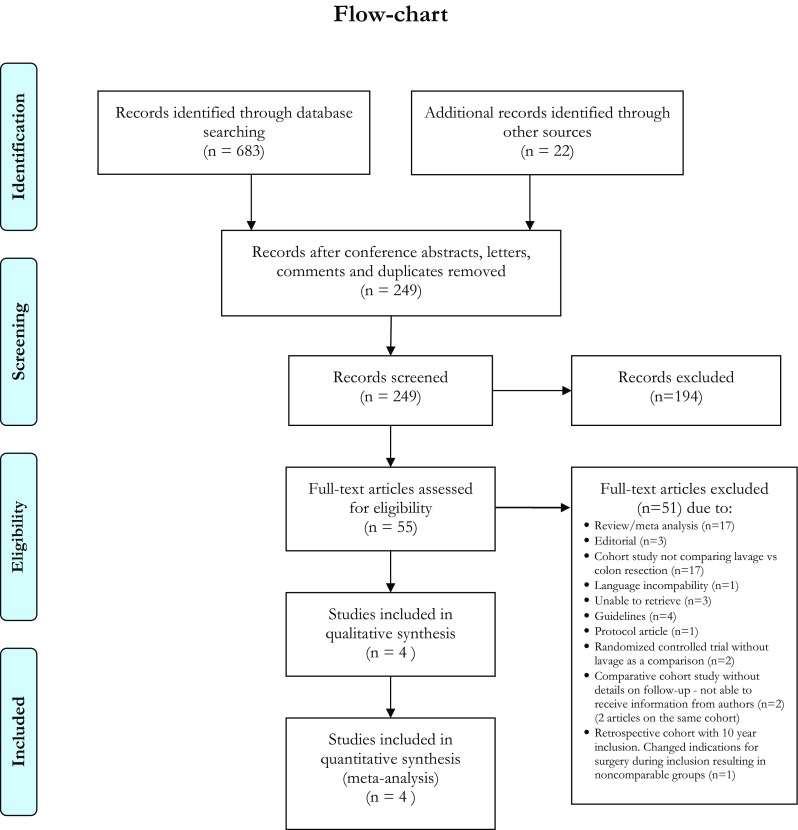
PRISMA diagram showing selection of articles for review

A PRISMA flow-chart shows the number of articles identified (*n* = 683) (Fig. 1). Only three randomized controlled trials were identified [12–15] as well as one cohort with controls [22]. Information on follow-up time was missing for the cohort study and it was therefore excluded from further analysis. Table 1 describes the study design characteristics of the included trials.

**Table 1:** Study design and assessment of the studies using the Grading of Recommendations Assessment, Development and Evaluation (GRADE) methodology [20]

Reference	Study arm	Hichey grade	No.	Random sequence generation	Allocation concealment	Blinding	Incomplete data outcome addressed	Risk of bias (limitation)	Directness (limitation)	Precision (limitation)
Vennix 2015 [15] “LOLA”	Laparoscopic lavage vs. Hartmann’s procedure or primary anastomosis	Only Hinchey grade III	88	Yes	Yes	No	Yes	Minor	No limitation	No limitation
Schultz 2015 [14] “SCANDIV”	Laparoscopic lavage vs. Hartmann’s procedure or primary anastomosis	Hinchey grade I–III	197	Yes	Yes	No	Yes	Minor	No limitation	No limitation
Thornell 2016 [13] “DILALA”	Laparoscopic Lavage vs. Hartmann’s procedure	Only Hinchey grade III	73	Yes	Yes	No	Yes	Minor	No limitation	No limitation

There were differences in the randomizing procedure and the control group: The LOLA and DIALA trials randomized after an initial diagnostic laparoscopy to ensure, as far as possible, that included patients had Hinchey III, whereas in SCANDIV randomization included all cases of diverticulitis scheduled for surgery. The LOLA trial randomized in two levels, first between laparoscopic lavage and resection surgery and at the second level between Hartmann’s procedure and colon resection with primary anastomosis with or without a diverting loop ileostomy. SCANDIV randomized between laparoscopic lavage and Hartmann’s procedure or colon resection with primary anastomosis with or without a diverting loop ileostomy; the type of resection surgery was at the discretion of the surgeon. The DILALA trial randomized between laparoscopic lavage and Hartmann’s procedure.

This render the patients in SCANDIV to differ from patients in LOLA and DILALA in that they may have a diverticulitis graded as Hinchey I-III (Hinchey IV was excluded per protocol before analysis). The resection groups in SCANDIV and LOLA differ from DILALA in the inclusion of two types of surgery: Hartmann’s procedure, colon resection with primary anastomosis with or without a diverting loop ileostomy, whereas DILALA only included Hartmann’s procedure as control.

Table 2 summarizes the patient characteristics and demography. As the protocols regarding the study cohorts were to some extent different, the studies became more comparable if only some of the specific reported analyses were used. From the LOLA trial, the modified intention to treat (ITT) was used [15]; from SCANDIV the primary intention to treat (ITT) was used [14]; and from DILALA, the per protocol analysis [13] was chosen.

**Table 2:** Patient characteristics and demography of included studies

		LOLA		SCANDIV		DILALA	
		Modified intention-to-treat		Primary intention-to-treat		Per protocol analysis	
		Laparoscopic lavage	Colon resection	Laparoscopic lavage	Colon resection	Laparoscopic lavage	Hartmann’s procedure
*n*		46	42	101	96	38	35
Sex	Male	26 (57 %)	25 (60 %)	44 (44 %)	45 (47 %)	20 (53 %)	14 (40 %)
Age	Mean (SD)	62.3 (12.7)	64.0 (12.3)	69.9 (13.5)	65.7 (15.2)	61.2 (16.5)	66.9 (12.8)
BMI	Mean (SD)	27.6 (6.2)	27.0 (4.4)	26.6 (4.9)	26.0 (4.4)	26.4 (2.5)	25.3 (4.3)
ASA I	*n* (%)	10 (22 %)	8 (19 %)	12 (12 %)	15 (16 %)	7 (18 %)	8 (23 %)
ASA II		21 (46 %)	13 (31 %)	47 (48 %)	37 (39 %)	21 (55 %)	12 (34 %)
ASA III		5 (11 %)	13 (31 %)	33 (33 %)	39 (41 %)	8 (21 %)	10 (29 %)
ASA IV		3 (7 %)	2 (5 %)	9 (9 %)	4 (4 %)	0	2 (6 %)
ASA V		0	0	0	1 (1 %)	0	0
Missing ASA						2 (5 %)	3 (9 %)
Previous diverticulitis	*n* (%)	12 (32 %)	10 (26 %)	19 (19 %)	24 (25 %)	5 (13 %)	5 (14 %)
Hinchey grade	I			3 (4 %)	2 (3 %)		
	II			1 (1 %)	4 (6 %)		
	III	46 (100 %)	42 (100 %)	70 (95 %)	64 (91 %)	38 (100 %)	35 (100 %)
Previous abdominal surgery		Missing	Missing	34 (34 %)	36 (38 %)	16 (42 %)	11 (31 %)

**Fig. 2:**
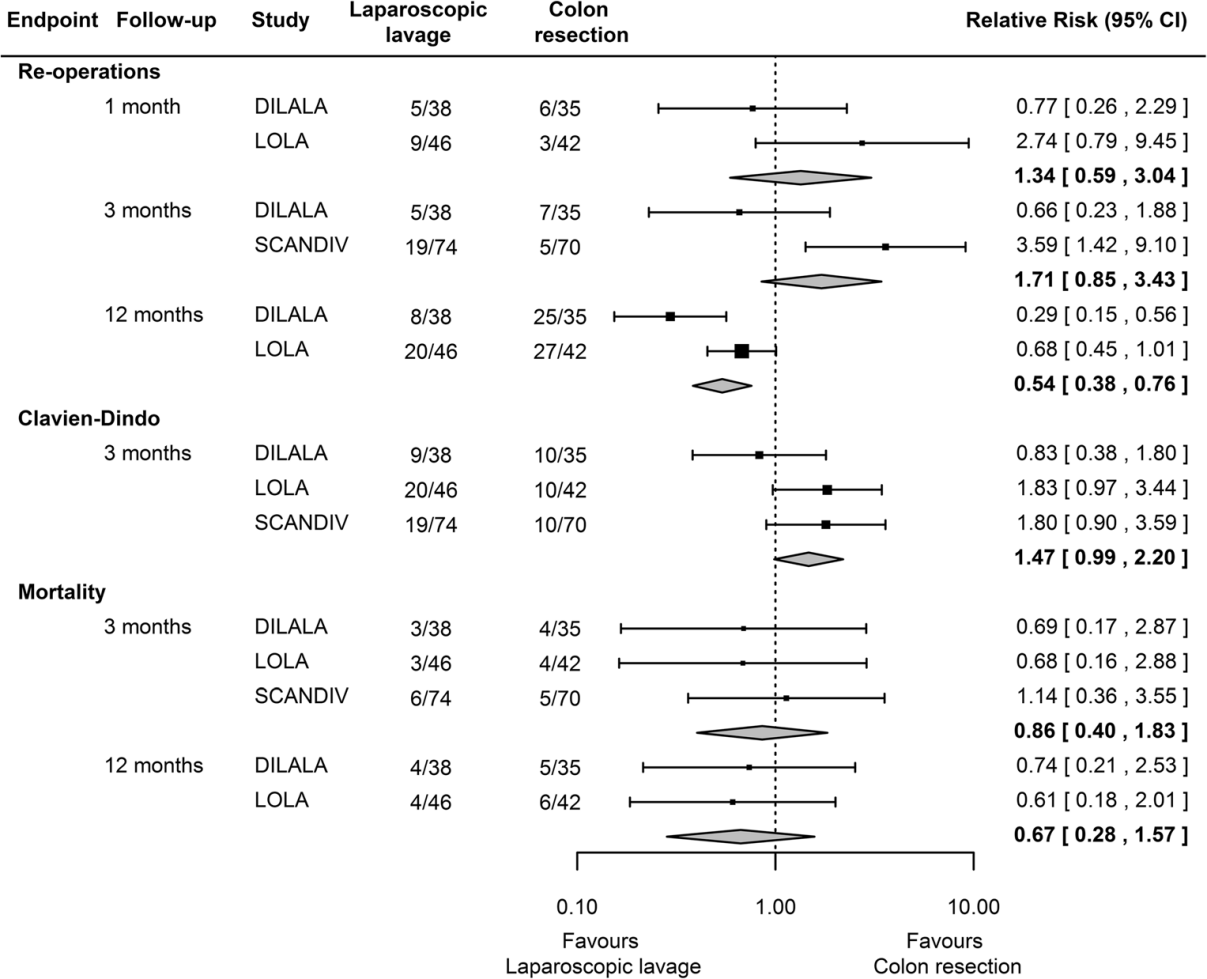
Forest plot comparing the effects of the two surgical techniques on re-operations, complications (according to Clavien-Dindo), and mortality. From: Moher D, Liberati A, Tetzlaff J, Altman DG, The PRISMA Group (2009). Preferred Reporting Items for Systematic Reviews and Meta-Analyses: The PRISMA Statement. PLoS Med 6(6): e1000097. doi:10.1371/journal.pmed1000097. For more information, visit www.prisma-statement.org

The combined estimate indicates a reduced risk for re-operation at 12 months among patients operated with laparoscopic to colon resection in the DILALA and LOLA trials (Fig. 2).

Results for the secondary endpoint complications at 90 days are shown in Fig. 2. There appear to be an increased risk for complications at least grade IIIB according to Clavien-Dindo for laparoscopic lavage compared to colon resection although this was not statistically significant. The combined estimate of mortality up to 3 months found a not statistically significant risk reduction for death for laparoscopic lavage compared to colon resection (Fig. 2). The same conclusions were made using the random effect model. The result for the random effect model and the assessment of heterogeneity is available in the supplement.

## Discussion

The basic findings of this systematic review and meta-analysis were that the need for re-operations within 12 months after index surgery was reduced after laparoscopic lavage compared to colon resection, with comparable morbidity and mortality. This finding combined with a recent detailed health economic analysis showing that Hartmann’s resection was overall more costly compared with laparoscopic lavage [16] supports the choice of laparoscopic lavage for this clinical condition.

Our study had both strengths and limitations. Before the search was initiated, a detailed search string was developed and a statistical analysis plan was finalized prior to analyses. Furthermore, the study was registered on the PROSPERO database [19] and is reported according to the PRISMA guidelines [18]. The availability of only three randomized studies is a limitation making assessment of heterogeneity difficult. The three randomized trials used in this meta-analysis were different in some aspects of their design. It seemed that LOLA [15] and SCANDIV [14] had more early complications after laparoscopic lavage compared with DILALA. The reason for this is unclear but may be due to differences between the studies regarding the patient cohorts. The age of patients was lowest in the DILALA trial which may translate into less comorbidity and thereby possibly a lesser risk for early complications. There were more patients with previous diverticulitis in LOLA and SCANDIV which also may affect results. The availability of interventional radiology for non-operative drainage procedures may also differ between hospitals and studies. These considerations are however hypothetical. Furthermore, there is a risk of bias as the choice of treatment in the control arm in SCANDIV was at the discretion of the surgeon. It is also possible that if primary anastomosis is a more favorable surgical procedure, the inclusion of both resection with a stoma and an anastomosis could provide better results for the colon resection group in both LOLA and SCANDIV. The health economic assessment looking at data from the DILALA trial showed results in favor of laparoscopic lavage, and the results were robust even when applying a sensitivity analysis with modification of variables 30 % in either direction [25].

In conclusion, the combined results of the three available randomized trials comparing laparoscopic lavage with colon resection in perforated purulent diverticulitis showed less need for re-operations within 12 months in favor of laparoscopic lavage after the index operation. Furthermore, there was no significant difference in severe morbidity or mortality between groups. Finally, as shown in a recent study, Hartmann’s resection was more costly than laparoscopic lavage, and it therefore seems advisable to perform laparoscopic lavage if technically feasible in patients with perforated purulent peritonitis on the basis of acute diverticulitis.
